# Phosphorus limitation enhances parasite impact: feedback effects at the population level

**DOI:** 10.1186/s12898-014-0029-1

**Published:** 2014-10-31

**Authors:** Katja Pulkkinen, Marcin W Wojewodzic, Dag O Hessen

**Affiliations:** Department of Biological and Environmental Science, University of Jyväskylä, P.O. Box 35, Jyväskylä, 40014 Finland; Department of Biosciences, University of Oslo, P. O. Box 1066 Blindern, Oslo, 0316 Norway; School of Biosciences, University of Birmingham, Birmingham, B15 2TT UK

**Keywords:** *Daphnia magna*, Ecological stoichiometry, Epidemiology, *Glugoides intestinalis*, Parasite, Transmission, Zooplankton

## Abstract

**Background:**

Nutrient deficiency affects the growth and population dynamics of consumers. Endoparasites can be seen as consumers that drain carbon (C) or energy from their host while simultaneously competing for limiting resources such as phosphorus (P). Depending on the relative demands of the host and the parasite for the limiting nutrient, intensified resource competition under nutrient limitation can either reduce the parasite’s effect on the host or further reduce the fitness of the nutrient-limited host. So far, knowledge of how nutrient limitation affects parasite performance at the host population level and how this affects the host populations is limited.

**Results:**

We followed the population growth of *Daphnia magna* that were uninfected or experimentally infected with a microsporidian, *Glugoides intestinalis*. The *Daphnia* were fed either P-sufficient or P-limited algae. The P-limited diet decreased the population density and biomass compared with the populations fed with the P-sufficient algae. In the P-sufficient populations, infection with the parasite reduced the population density but not the biomass of *Daphnia*, while in the P-limited populations, both the density and biomass of *Daphnia* decreased toward the end of the 32 day experiment compared with the uninfected controls. The infected animals from the P-limited populations had higher parasite spore cluster counts, while, in a separate experiment, host diet quality did not affect the number of parasites in individually kept *Daphnia*.

**Conclusions:**

Because host diet quality did not affect parasite numbers at the individual level, we suggest that the higher parasite load in the P-limited populations is a result of feedback effects arising at the population level. Because of the density-dependent transmission of the parasite and the time lag between exposure and transmission, the lower host population density in the P-limited populations led to a higher spore:host ratio. This effect may have been further reinforced by decreases in filtration rates caused by crowding in the P-sufficient populations and/or increases in filtration rates as a response to poor food quality in the P-limited populations. The increases in exposure led to a higher parasite load and aggravated the negative effects of parasite infection at the population level.

**Electronic supplementary material:**

The online version of this article (doi:10.1186/s12898-014-0029-1) contains supplementary material, which is available to authorized users.

## Background

The production and biomass of organisms are affected by the abundance and quality of available resources (i.e., bottom-up control) and predators (i.e., top-down control) [1]. In aquatic ecosystems, fish and invertebrate predators have attracted the most interest in food web studies, while the role of parasites in food webs has been largely neglected despite their pervasiveness and importance. Parasite “predation” and interaction with its host’s nutritional status can be far subtler than the direct impact of fish predation.

Parasites drain energy from their hosts, thereby affecting their hosts’ growth, reproduction and survival and, consequently, the population dynamics of the hosts [2]. Traditionally, heavy parasite burdens have been interpreted as a sign of poor host condition, while hosts in good condition may escape serious infections because they can afford to invest in immune responses or other defences against parasites [2–4]. This view is, however, challenged by experiments demonstrating that parasites can perform better in well-fed hosts [5–9]. Generally, the resources acquired by the parasite are considered in terms of energy, i.e., the resource quantity in terms of carbon (C) [10,11], while the role of elemental nutrients such as nitrogen (N) or phosphorus (P) as limiting resources for parasite growth has received less attention [10,12].

The inclusion of elemental ratios (e.g., C:P) in models may generate substantially different population dynamics in prey and predator systems compared with previous models that considered only resource quantity [13]. When the relative element content of the food (e.g., C:P) is below the threshold elemental ratio (TER, the minimum level needed for somatic maintenance) of the consumer, there will be C in relative excess, and consumer C-use efficiency and growth rate will decrease even when food is abundant [13]. Thus, at high food abundance but low food quality, consumers will suffer from “quality starvation” [14].

Interactions with other consumers, such as predators or parasites, may further complicate the consumer population response to food quality, and, in fact, resource competition theory has been suggested as a basis for predicting the outcomes of infectious diseases [12,15,16]. According to this theory, hosts and pathogens compete for the resources (and not necessarily energy or C) that the host uses for its own growth and maintenance in a pathogen-free situation [12,15,16]. “Quality starvation” of hosts would be expected to increase the strength of competition for limiting elements between hosts and parasites, and the net outcome of this enhanced competition would depend on the demands of the host and the parasite for the limiting nutrient [12]. Thus, a low quality diet might, despite reducing the fitness of the host, also decrease the relative effect of parasites. Alternatively, the increased drain of nutrients caused by parasites might further amplify the effect of nutrient limitation on hosts and lead to decreased host reproduction, as well as increased mortality [17]. Correspondingly, parasite demands will determine the effect of host resources on parasite growth. Understanding the interactions between parasites and food quality is essential to predict the outcomes of infectious diseases in the face of changing nutrient loading [18] and whether focus at the individual or population level may yield different predictions.

We studied how host nutrient limitation combined with parasite infection affected the host and the parasite populations in a model system consisting of a freshwater crustacean, *Daphnia magna*, and its microparasite. In aquatic environments, *Daphnia* often play a key role in mediating effects between primary producers and secondary consumers [19] and, consequently, in the recycling of nutrients [20]. *Daphnia* have a high demand for P [21] and have an ability to maintain a high degree of homeostatic control [22]. Because *Daphnia* commonly feed on P-limited seston (i.e., food with a high C:P ratio), they are likely to face P limitations on growth in nature [22–24]. *Daphnia* populations in nature are frequently parasitised by various species of bacterial, fungal and helminth parasites, with negative effects on population density [25–28]. At the individual level, host nutrient limitation has been shown to decrease growth and production of infective propagules for bacterial [29] and fungal [30] parasites of *Daphnia*. However, effects detected at individual level might not always predict the outcome of host-parasite interaction at population level [31]. At present, knowledge is sparse on how nutrient limitation affects parasite performance at the host population level or how this affects the host populations.

## Results

### Population dynamics

**Figure 1:**
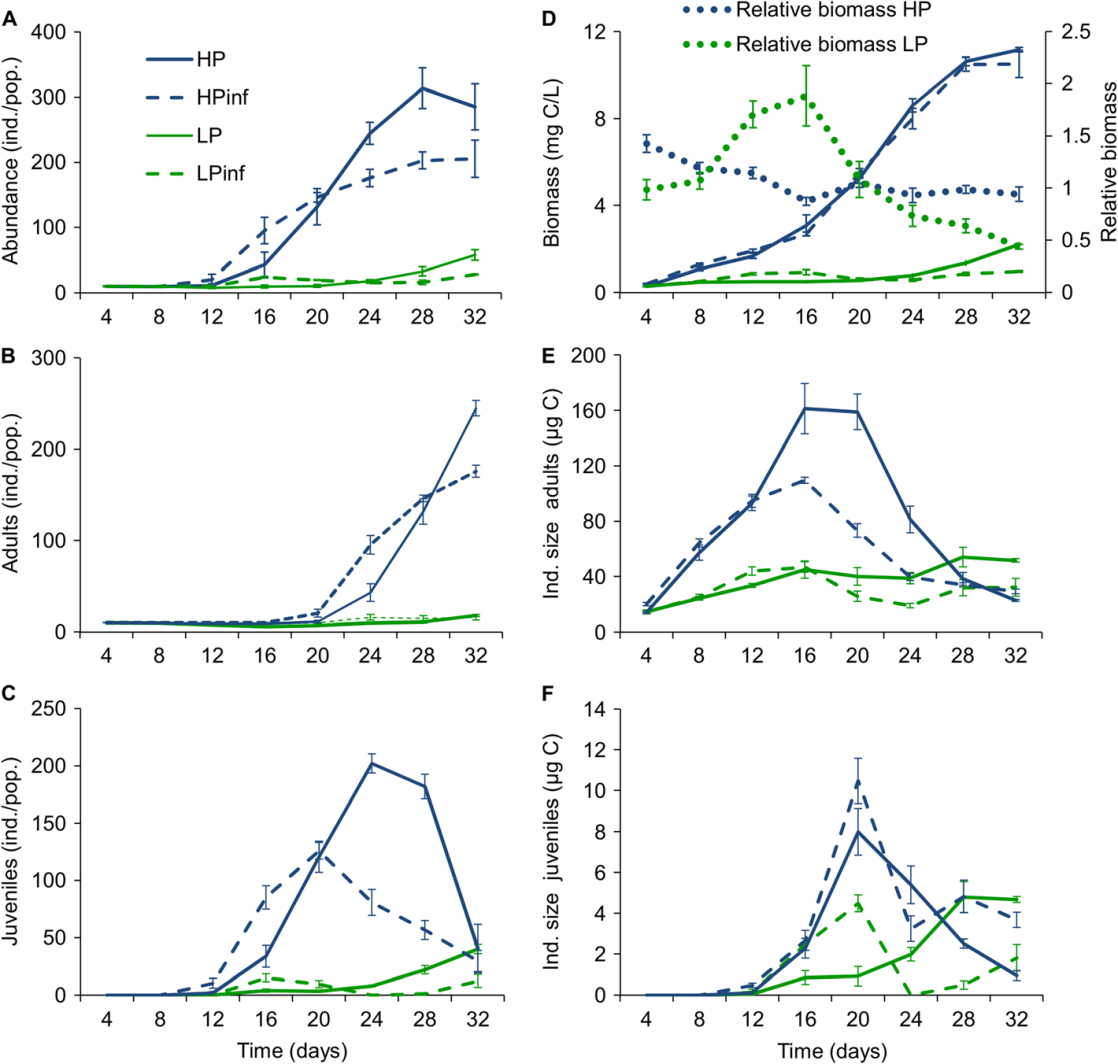
**Abundances and biomasses in different treatments in the 32 day experiment. (A)** Mean total **(B)** adult and **(C)** juvenile abundances (± SE) of *Daphnia magna* in uninfected control populations fed with P-sufficient (HP, denoting for high P) or P-limited (LP, denoting for low P) green algae and in populations infected with a microsporidian *Glugoides intestinalis* (HPinf and LPinf), **(D)** Mean carbon biomass mg C L^-1^ (± SE) in all four treatments and relative biomass (± SE) of the infected populations for the P-sufficient (HP) and P-limited (LP) treatments during the experiment, **(E)** Mean individual size of adult, and **(F)** juvenile *Daphnia* μg C (± SE). The populations were started with 10 juveniles each born to uninfected mothers fed with P-sufficient algae, and half of the animals were infected before allocation to feeding treatments. See Materials and methods for calculation of relative biomass. Note that the abundances are given per population in 0.5 L vials to emphasise the actual densities, while biomass is given per L to aid comparison with previously published results. The legend in panel **(A)** applies to treatments in all panels **(A)** - **(F)**, while the legend in panel **(D)** indicates the relative biomass in panel **(D)**.

During the 32-day experiment, the population numbers peaked at approximately 300 individuals after 28 days in the uninfected populations fed with the P-sufficient algae (Figure 1A). For the infected populations fed with the P-sufficient algae, the numbers reached a lower density of approximately 200 individuals compared with the uninfected populations (Figure 1A; Table 1, within-subject effects, significant interaction between day (D) and infection status (I); Table 2, within subject contrasts for D × I). The uninfected populations fed the P-limited food reached a maximum density of approximately 65 individuals, while the infected P-limited populations remained at a lower level of fewer than 40 animals (Figure 1A; Tables 1 and 2, within subject contrasts). In the P-sufficient treatments, the numbers of adults increased towards the end of the experiment. In the infected populations, this occurred sooner but ended at a lower level compared with the uninfected populations (Figure 1B; Tables 1 and 2, within subject contrasts). Juvenile abundance in the P-sufficient treatments peaked between days 20 and 24 and decreased thereafter, and this decline started sooner in the infected populations (Figure 1C; Tables 1 and 2 within subject contrasts). In the P-limited treatments, the numbers of adults did not differ between the infected and the uninfected populations (Figure 1B; Table 1). The abundance of juveniles in the P-limited populations was at first higher in the infected than in the uninfected populations. In the uninfected P-limited populations the numbers of juveniles declined to zero, followed by a new increase at day 32, while in the infected populations the numbers increased more steadily (Figure 1C; Tables 1 and 2, within subject contrasts).

**Table 1:** **Results of the repeated measures ANOVAs for** ***Daphnia*** **population data**

			**P** **-** **sufficient**			**P** **-** **limited**		
			**df**	**F**	**p**	**df**	**F**	**p**
Abund. total	BSE^a^	Infection (I)	1	0.59^c^	0.473	1	0.01^c^	0.934
		Error	6			6		
	WSE^b^	Day (D)	5	274.5	<0.001	5	53.9	<0.001
		D x I	5	15.7	<0.001	5	24.4	<0.001
		Error	30			30		
Abund. adults	BSE	Infection (I)	1	5.59^c^	0.056	1	2.90^d^	0.140
		Error	6			6		
	WSE	Day (D)	3	239.8	<0.001	1.05^e^	3.18	0.122
		D x I	3	11.6	<0.001	1.05^e^	1.88	0.218
		Error	18			6.27^e^		
Abund. juv.	BSE	Infection (I)	1	10.55^f^	0.017	1	17.7^f^	0.006
		Error	6			6		
	WSE	Day (D)	1.98^e^	24.7	<0.001	4	16.9	<0.001
		D x I	1.98^e^	14.2	<0.001	4	20.1	<0.001
		Error	11.9^e^			24		
Biomass	BSE	Infection (I)	1	0.26	0.631	1	2.9	0.139
		Error	6			6		
	WSE	Day (D)	2.52^e^	425.0	<0.001	2.85^e^	74.1	<0.001
		D x I	2.52^e^	0.88	0.529	2.85^e^	32.6	<0.001
		Error	15.1^e^			17.1^e^		
Ind. size adults	BSE	Infection (I)	1	30.4^g^	<0.001	1	16.18^f^	0.007
		Error	6			6		
	WSE	Day (D)	2.24^e^	175.1	<0.001	2.13^e^	16.3	<0.001
		D x I	2.24^e^	15.0	<0.001	2.13^e^	4.7	<0.001
		Error	13.5^e^			12.8^e^		
Ind. size juv.	BSE	Infection (I)	1	5.4	0.059	1	11.8^f^	0.014
		Error	6				6	
	WSE	Day (D)	5	46.2	<0.001	5	22.5	<0.001
		D x I	5	4.4	0.004	5	29.2	<0.001
		Error	30			30		

Tested variables are population abundance (Abund. total), abundances of adults and juveniles, population biomass (mg C L^-1^) and individual sizes (μg C) of adults and juveniles for uninfected and infected *Daphnia magna* in P-sufficient and P-limited populations in the 32-day experiment.

^a)^Between subjects effects, ^b)^Within subject effects, ^c)^ln-transformed, ^d)^transformation y = -1/(x + 1)^3, ^e)^Greenhouse Geisser corrected statistics, ^f)^square root-transformed, ^g)^transformation y = ln(x + 1).

**Table 2:** **Within**-**subject contrasts for repeated measures ANOVA**

		**Abundance**				**Individual size**	
		**Total**	**Adult**	**Juv.**	**Biom.**	**Adult**	**Juv.**
P-sufficient	Day 8 vs. Day 4				0.385	0.055	
	Day 12 vs. previous				0.593	0.028	
	Day 16 vs. previous	0.300			0.280	0.001	0.966
	Day 20 vs. previous	0.032		0.018	0.542	0.001	0.237
	Day 24 vs. previous	<0.001	0.251	0.001	0.289	0.018	0.028
	Day 28 vs. previous	0.001	0.032	0.001	0.835	0.468	0.063
	Day 32 vs. previous	0.004	<0.001	0.476	0.401	0.002	0.035
P-limited	Day 8 vs. Day 4				0.414	0.656	
	Day 12 vs. previous				0.001	0.027	
	Day 16 vs. previous	0.018			0.067	0.793	0.009
	Day 20 vs. previous	0.780	0.284	0.043	0.119	0.033	0.001
	Day 24 vs. previous	0.001	0.248	0.001	0.007	0.017	<0.001
	Day 28 vs. previous	<0.001	0.178	0.001	0.006	0.129	0.001
	Day 32 vs. previous	0.008	0.087	0.058	<0.001	0.150	0.023

Within-subject contrasts for the interaction between day (D) and infection (I) for the repeated measures ANOVA on Daphnia population data presented in Table 1. The contrast is “difference”, which compares each level to the mean of previous levels.

A carbon content of 44 ± 0.02% (mean ± SE) dry weight was found for uninfected animals in the P-sufficient treatment at the end of the 32 day population experiment and was used in subsequent biomass calculations. The populations fed with P-sufficient algae yielded maximum biomasses of 10.5 to 11.2 mg C^-1^ L^-1^, with no significant interaction detected between the uninfected and the infected populations over time (Figure 1D; Table 1, within-subject effects, D × I). In the populations fed with the P-limited algae, biomasses remained below 2.2 mg C^-1^ L^-1^ (Figure 1D), and there was a significant difference between the uninfected and the infected populations in biomass accumulation over time; the infected populations had increasingly lower biomasses than the uninfected populations from day 24 until day 32 (Figure 1D; Tables 1 and 2, within subject contrasts).

The overall effect of infection on population biomass is illustrated in the relative biomasses of the infected populations compared to those of the uninfected ones (Figure 1D). For the P-sufficient treatment, the relative biomass remained at approximately 0.8 to 1.0 for the entire experimental period (one-sample t-tests, p > 0.05). The P-limited treatment experienced higher variability (Figure 1D), and at the end of the experiment, infection led to a dramatic decline to less than half of the biomass of the uninfected populations (one-sample t-tests, for days 4-28 p >0.05, for day 32 Bonferroni-corrected p = 0.002).

In the P-sufficient treatment, adults were larger in the uninfected than in the infected populations (Figure 1E; Table 1, between subjects effects for I), but significant D × I interactions indicated that this difference became apparent only after the second generation became adults (day 16; Figure 1B,E; Tables 1 and 2). Juvenile size in the P-sufficient populations was lower for the uninfected than the infected animals on days 28 and 32 (Figure 1F; Tables 1 and 2, within-subject contrasts). In the P-limited populations, both adults and juveniles were at first larger in the infected than in the uninfected populations (Figure 1E,F; Tables 1 and 2, within-subject contrasts, days 12 to 16 for adults, days 12 to 20 for juveniles), but the situation reversed after day 20 (Figure 1E,F; Tables 1 and 2, within-subject contrasts).

**Figure 2:**
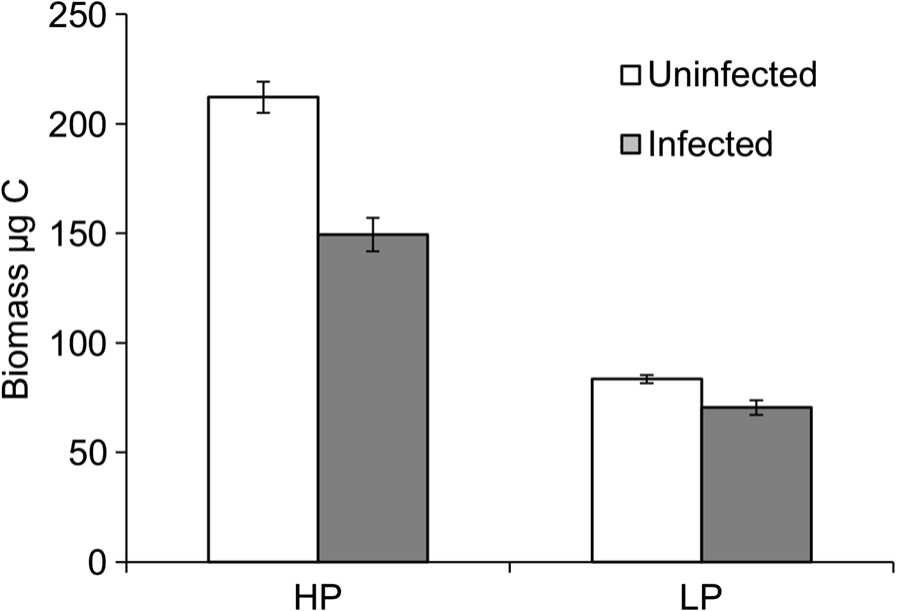
**The mean sizes of founder animals in the 14 day experiment.** The mean carbon biomasses mg C L^-1^ (± SE) of the uninfected and infected founder animals of equal age fed with the P-sufficient (HP) or the P-limited (LP) algae at the end of the 14 day experiment.

In the 14-day experiment, population growth was slightly higher than in the 32-day experiment (Figure 2), but the differences among treatments confirmed the pattern observed in the 32-day experiment. At the end of the 14-day experiment, the mean abundances of animals did not differ between the infected and the uninfected populations in either of the feeding treatments (Table 3), but the numbers were significantly higher in the P-sufficient populations (182 ± 6 and 178 ± 15 (mean ± SE) for uninfected and infected *Daphnia*, respectively) than in the P-limited treatments (25 ± 4 and 24 ± 1 for uninfected and infected *Daphnia*, respectively, Table 3). The mean size of the founder animals fed with P-sufficient food was higher than for those fed with P-limited food and higher for uninfected than infected animals (Figure 2, Table 3). The difference in size between the uninfected and the infected animals was larger for animals fed with the P-sufficient food (Table 3).

**Table 3:** **Population abundance and mean size in the 14 day population experiment**

		**df**	**F**	**p**
Population abundance	Food (F)	1	352.2	<0.001
	Infection (I)	1	0.12	0.736
	F x I	1	10.6	0.848
	Error	12		
Mean biomass of founder animals	Food (F)	1	352.3	<0.001
	Infection (I)	1	47.0	<0.001
	F x I	1	20.3	<0.001
	Error	12		

Two-way ANOVAs for the population abundance and the biomass (mg C L^-1^) of the uninfected and infected founder animals at the end of the 14-day experiment for *Daphnia magna* in P-sufficient and P-limited populations.

### Parasite load

**Figure 3:**
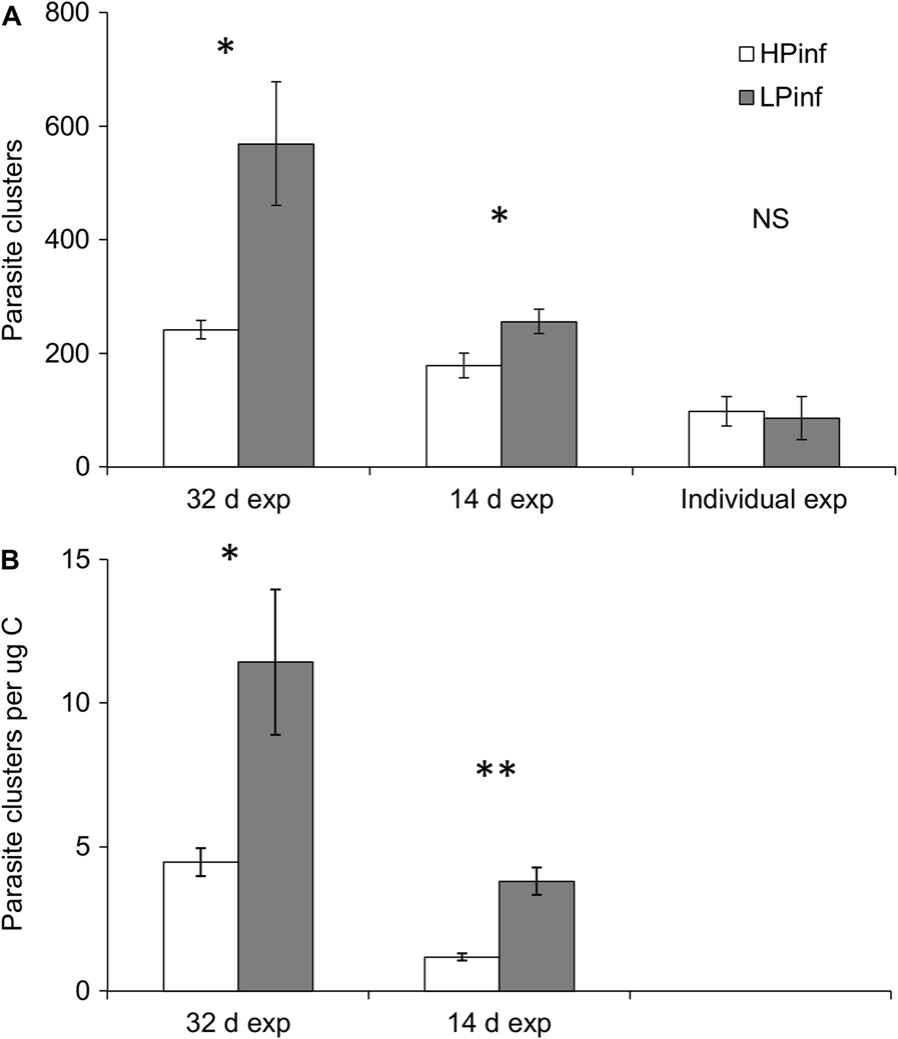
**Parasite load in the different feeding treatments. (A)** The number of *Glugoides intestinalis* spore clusters in the gut of *Daphnia magna* (mean ± SE) for animals fed with the P-sufficient food (HP inf) and for animals fed with the P-limited food (LP inf) at the end of 32 day and 14 day population experiments, and in animals exposed individually before allocation to the two feeding treatments. **(B)** The number of spore clusters (mean ± SE) per *Daphnia* biomass (μg C) in the P-sufficient (HP inf) and the P-limited (LP inf) treatments at the end of 32 day and 14 day population experiments. Pairwise comparisons between treatments within different experiments: *P <0.05, **P <0.01, NS not statistically significant.

At the end of the 32-day experiment, the mean number of parasite clusters in adult *Daphnia* was 569 ± 109 (mean ± SE) for *Daphnia* fed with the P-limited food and was significantly greater than the mean of 241 ± 16 for *Daphnia* fed with the P-sufficient food (Figure 3A; t-test t_6_ = 2.97, p = 0.025). The mean parasite load per carbon biomass of *Daphnia* was significantly higher for the animals fed with the P-limited food than for animals fed with the P-sufficient food (Figure 3B; ANOVA, exp-transformed values, F_1,6_ = 13.54, p = 0.010). Animals dissected from uninfected populations were found to be free of parasites.

At the end of the 14-day experiment, in founder animals of the same age, the mean number of parasite clusters was significantly higher for *Daphnia* fed with the P-limited food (256 ± 22; mean ± SE) than for *Daphnia* fed with the P-sufficient food (179 ± 21; Figure 3A; t-test, t_6_ = 2.55, p = 0.044). The mean parasite load per carbon biomass of *Daphnia* was significantly higher for the animals fed with P-limited food than for animals fed with P-sufficient food (Figure 3B; t-test, t_6_ = 5.35, p = 0.002). No parasites were detected from animals dissected from uninfected populations.

In animals that were exposed pairwise to the same donors and allocated singly to different food treatments, the mean numbers of parasite clusters did not differ between animals fed with the P-limited (98 ± 38; mean ± SE) or the P-sufficient (86 ± 26) diet (Figure 3A; paired t-test t_20_ = 0.29, p = 0.772). The number of spore clusters in animals fed with the P-sufficient or the P-limited algae did not correlate with each other (Spearman correlations, p >0.05). The number of spore clusters in donor females did not correlate with the number of spore clusters in the experimental *Daphnia* fed with the P-limited algae, but the correlation was positive for animals fed with the P-sufficient algae (Spearman’s’ rho = 0.61, p = 0.003, n = 21).

## Discussion

The results of our experiments demonstrated that infection by the microsporidian *Glugoides intestinalis* exerted a strong check on the population density of *Daphnia*, regardless of food quality, in agreement with the results of earlier studies [5,31,32]. Over time, however, population biomass was more severely affected by the parasite under P-limited conditions, clearly suggesting that the infection posed an extra burden on animals suffering from dietary nutrient deficiency. Contrary to expectations based on results from other *Daphnia*-parasite systems [29,30], animals collected from the P-limited populations had higher numbers of parasite spore clusters than animals from the P-sufficient populations. An additional experiment indicated that food quality did not affect the development of spores. This result suggests that the greater number of spore clusters in the P-limited populations is the result of population level phenomena arising from feedback effects between spore production and exposure in host populations.

In the early phase of the experiment, the infected populations produced more offspring than the uninfected populations. For *Daphnia*, a life history shift towards producing more offspring earlier in life could compensate for decreases later in life caused by parasitism. Such a plastic response has been previously demonstrated for *D. magna* exposed to *G. intestinalis* [33]. However, during the course of the experiment, densities in the infected populations decreased due to hampered reproduction (Figure 1A-C) and/or increased juvenile mortality. In the P-limited populations, the population biomass decreased to 50% in the infected populations relative to the uninfected populations by the end of the experiment. Our results suggest that under severe P limitation the additional burden from parasites intensifies the negative population impacts of nutrient limitation, agreeing with previous reports on the combined effects of nutritional stress and parasite infection on hosts [2,4].

According to the individual level experiments, where individual *Daphnia* juveniles were first exposed to a single dose of parasite spores and then reared either under P-sufficient of P-limited diet, host food quality did not directly affect the development of *G. intestinalis* spore clusters within the hosts. In other host-parasite systems, e.g. a fungal parasite in a diatom host [9] and a bacterial parasite in *Daphnia* [29], host P-limitation has been shown to affect parasite growth negatively. *G. intestinalis* does not seem to be limited by P, but possibly by some other nutrient or C [34].

**Figure 4:**
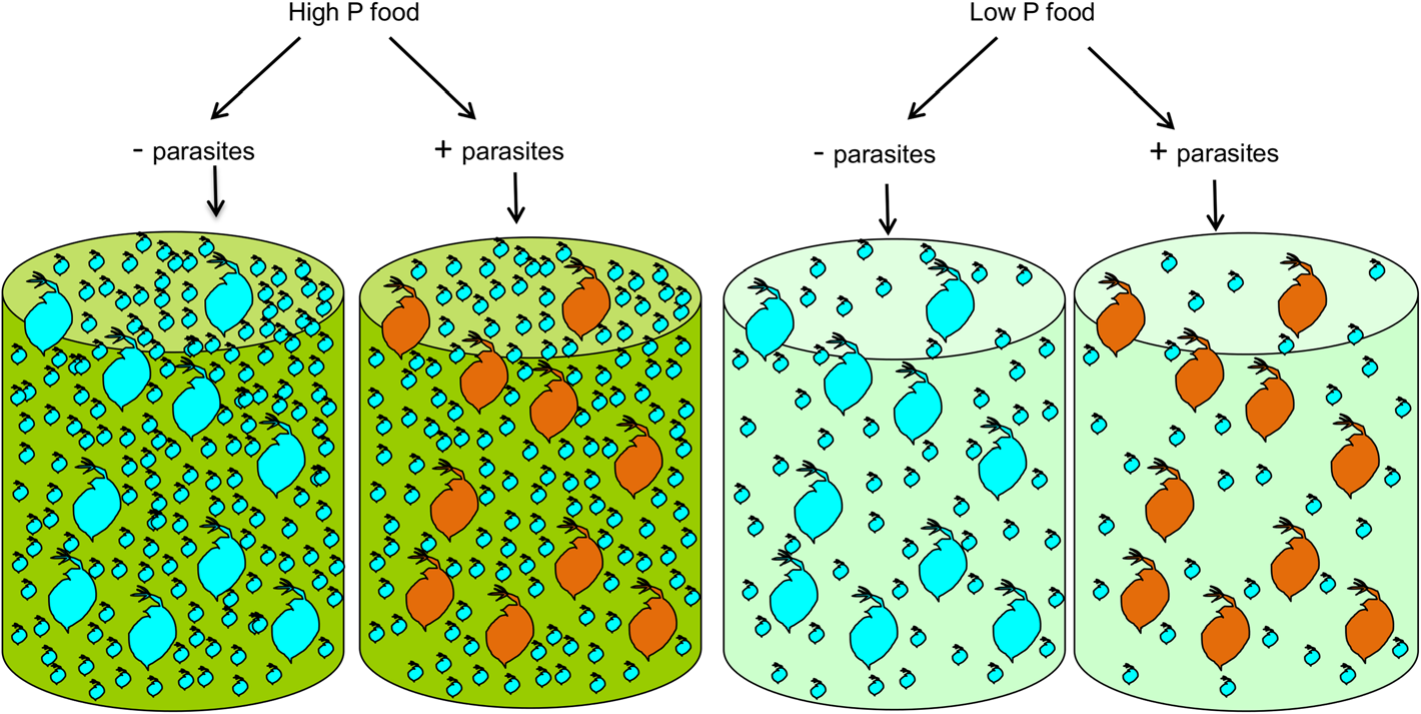
**Conceptualisation of population responses and per capita parasite infection related to diet treatment.** The P-limited diet (light green jars on right) led to a five- to six-fold decrease in population numbers of *Daphnia magna* compared to populations receiving P-sufficient food (green jars on left) but did not affect the spore production in infected founder animals (depicted as orange *Daphnia*). Because of a 10-14 day time lag in parasite development, the density of juveniles, not yet producing spores but filtering spores from water, remained lower in the P-limited than in the P-sufficient populations, leading to a higher per capita spore load in the P-limited *Daphnia* populations.

As parasite within-host growth did not differ between P-sufficient and P-limited hosts, the difference in parasite loads between the two feeding treatments might have simply reflected differences in the per capita exposure rates to spores because of differences in population densities. The transmission of *G. intestinalis* is density-dependent, and increased per capita exposure to spores increased the probability of infection [35]. Because of the 10-14 day lag between exposure and the onset of spore production [36], most of the spores in the populations during the first three weeks were produced by the founder animals, which were exposed in the initial phase of the experiment. In the P-limited populations, the density of juveniles that were not yet producing spores themselves but filtering spores from the water was lower than in the P-sufficient populations. Consequently, in the P-limited populations, spores were shared among fewer animals than in P-sufficient populations, leading to higher number of spore clusters per animal in the P-limited populations. The P limitation thus led to higher parasite numbers by increasing the per capita exposure rates to parasite spores via density effects at the host population level (Figure 4).

Additionally, changes in filtration rates could affect the intake of spores from the water [5], as well as their release into the water from the gut [37]. Crowding in the P-sufficient populations might have decreased their filtration rates [38], while low food quality in the P-limited population might have increased their filtration rates [39,40]. Indeed, water from the crowded *Daphnia* populations has earlier been shown to reduce transmission of *G. intestinalis* [41]. On the other hand, previous experiments with the *Daphnia* clone used in this study found five-fold lower ingestion rates for animals grown with the P-limited algae than for those fed with the P-sufficient algae [34]; thus, the responses of animals subjected continuously to food quality limitations might differ from those obtained with short-term changes in the diet as performed in the ingestion rate experiments [39,40]. While the differences in the spore loads in the 32-day experiment may have been confounded by age and size, in the 14-day experiment all *Daphnia* screened for parasites were the founders of populations of the same age and had equal exposure time for infection. Our results are in agreement with studies showing a “dilution” effect, where high host density or high diversity of hosts and/or non-hosts can remove parasite propagules, which can lead to a decrease in parasite spread [41–45].

To reveal the complex interactions between host and parasite under various P-regimes, pools and mass balance for C and P should be studied for both the host and the parasite. Resolving the effect of P on parasite fitness would require measurements of transmission success as well as infectivity of the spores to the next host. Additionally, the relationship between parasite loads and available nutrients should be further studied using a wider array of host clones to reveal possible genotype x environment interactions [46,47].

The population experiments were performed within a confined space with donor-controlled algal dynamics, i.e., the algal stock was replenished in cultures every second day. This setup differs from a situation where the algal C:P dynamic is governed by the interaction between grazing and recycling of nutrients by herbivores [20]. The uninfected *Daphnia* dynamics in our batch-type setup resemble those obtained in two-step flow-through chemostats [14,48]. However, parasite spore availability might have been different in a flow-through system, where a wash out of spores is possible. Although the phenomenon described here might be transient, it could operate within populations in closed systems, such as in experimental settings, and could be important in natural ecosystems, especially in ponds and small lakes. Previously, Frost *et al*. [29] reported reduced pathogen production, and Penczykowski *et al*. [49] reported reduced transmission potential in individual hosts receiving low-quality food; both reports suggested that reductions in disease incidence could occur in ecosystems limited by poor resource quality. Our results indicate that through its effects on population dynamics, nutrient limitation may increase parasite exposure and aggravate the negative effects of parasite infection at the population level.

## Conclusions

For the host-parasite combination used in the current study, the additional burden from parasites intensified the negative population impacts of nutrient limitation. Furthermore, our results suggest that nutrient limitation affected parasite transmission and exposure via its effect on host population dynamics rather than by directly affecting parasite growth within the host.

## Methods

### The host-parasite system

The model system used in the experiment consisted of *Daphnia magna* Straus (Crustacea: Cladocera) and a microsporidian *Glugoides intestinalis* (Microspora: Glugeidae) [50]. The *Daphnia* were obtained from the laboratory of Prof. Dieter Ebert (Basel, Switzerland). The nonselective filter feeding of *Daphnia* makes them vulnerable to infection by parasites that release infective propagules the size of *Daphnia* food particles into the water. *G. intestinalis* is an intracellular parasite in the gut epithelium of *Daphnia* that infects its host through waterborne spores. Inside the host cell, it undergoes sporogony and produces spherical clusters of spores [35]. It reproduces directly and transmission is horizontal between hosts [5]. Hosts do not recover from infection [31]. The parasite is transmitted from live hosts, and reinfection of the same host is common when spores released in faeces are ingested during filter feeding [5]. The *Daphnia* clone used in the experiments is the original host for the parasite and because of high transmissibility and the equal susceptibility of the hosts, prevalence within a homogeneously mixed population can be expected to reach 100%. Moreover, the transmission is density-dependent [4], and the number of parasite spores found inside the host is directly correlated with the parasite’s transmission success [32] so that exposure to larger doses (higher concentrations of the waterborne spores) leads to higher parasite loads [35]. Spore clusters become visible in the gut after approximately one week, but their numbers increase rapidly after approximately 10-14 days [5,36].

### Population dynamics experiment

The food algae, *Pseudokirchneriella subcapitata* Korschikov, was grown in continuous chemostats in sterile COMBO medium [51] as described by Wojewodzic *et al*. [52]. The P-sufficient algae were grown in medium containing 50 μM of P, at a dilution rate of 0.2 d^-1^, yielding a stable molar C:P ratio of 100. For the P-limited algae, the P concentration was reduced to 2 μM of P, which resulted in C:P ratios that ranged between 700-1000. Subsamples of algae were filtered on pre-ignited (500°C, 4 h) GF/F filters and analysed for particulate C in a Carlo Erba CHN 1106 elemental analyser. Particulate P was measured on corresponding filters placed in 5 mL of distilled water supplemented with peroxodisulfate (0.05 g K_2_S_2_O_8_), which were then autoclaved (121°C, 1 h). P content was then analysed by the molybdate-blue method [53].

Prior to the experiment, the stock cultures of *Daphnia* were kept in glass jars filled with 2 L of P-free COMBO medium and fed *ad libitum* with a suspension of the food algae grown in batch cultures in standard-P COMBO medium at 20°C. All glassware used for growing zooplankton was cleaned with soda and acid and rinsed with distilled water to avoid C and P contamination.

The experiments were started with neonates (<24 h) originating from at least the second maternal brood. The neonates were distributed randomly in groups of ten to 80 mL of P-free COMBO. Half of the neonates were exposed to parasite infection by cohabitation with five *D. magna* females infected with *G. intestinalis* for 24 h. This exposure time leads to 100% infection of the neonates [41]. The control animals were treated similarly, with females collected from uninfected cultures. The animals were supplied with the P-sufficient algae at 2 mg C L^-1^. After 24 h, the juveniles were transferred into glass jars filled with 500 mL of fresh P-free COMBO, such that juveniles exposed to parasite infection were distributed randomly to each of eight populations and uninfected juveniles to each of eight control populations.

Half of both the infected and the control populations were randomly allocated to two feeding treatments with either P-sufficient algae or with P-limited algae, resulting in four replicate populations for each of the parasite-feeding treatment combinations (see Additional file 1 for schematic representation). *Daphnia* in both treatments received 0.5 mg algal C L ^-1^ day ^-1^ on the first day, 1 mg C L ^-1^ day ^-1^ on the third day and subsequently 2 mg C L ^-1^ day ^-1^. The populations were transferred to fresh media and fed every second day. Every fourth day, the animals were photographed and analysed using the image analysis technique described by Faerovig *et al*. [54] to measure population size and biomass. The ellipsoid volumes or lengths measured from photographs with the ImageJ software (http://imagej.nih.gov/ij, version 1.42q) were converted to biomass using pre-established relationships for uninfected P-sufficient animals: dry weight (mg) = 0.25 × volume (μm^3^)^0.91^, r^2^ = 0.91; and dw (mg) = 12.64 × length (mm)^2.73^, r^2^ = 0.90. The experiment was terminated after 32 days, when the population density began to decline in the uninfected P-sufficient treatment. Then, six to ten large adults were collected from each population and placed into separate jars with fresh COMBO for determination of parasite clusters [35]. Additional animals were collected from the P-sufficient populations for analysis of C content.

At the end of the first experiment, the animals collected for determination of parasite abundance varied in age, which may have affected the parasite load. To verify the parasite load from the different food treatments in the first experiment, we conducted a second experiment that was terminated at day 14, when the parasite load could be compared in founder animals that were of the same age. The populations were initiated as described above for the first experiment. At the end of the experiment, all remaining founder animals (8-10 per population) were collected for determination of parasite clusters.

### Individual exposures

To test whether food quality affected the formation of spore clusters within individual *Daphnia*, pairs of *Daphnia* juveniles were exposed to infection under similar conditions and were fed with algae of the two different food qualities. The *Daphnia* were maintained in an artificial freshwater medium [55] modified as in [41]. Neonates (<24 h) originating from at least the second brood of mothers were collected randomly two at a time and were placed in 100 mL of medium with two females infected with *G. intestinalis*. After 24 hours, the juveniles were transferred to clean medium. One juvenile from each pair was randomly assigned to the P-sufficient algae or to the P-limited algae feeding treatment. The animals were fed at least 2 mg C L ^-1^ day ^-1^ and were transferred to fresh medium every second day. The numbers of spore clusters were examined after 12 days, according to Ebert & Mangin [35]. The *Daphnia* were fed with green algae *Scenedesmus gracilis* grown in 4 L batch cultures in WC-medium [56] without vitamins. The P-sufficient algae were grown in medium containing 50 μM of P and the P-limited algae in medium containing 5 μM of P. The P-sufficient cultures were diluted to half of the volume twice (0.14 d^-1^), and the P-limited cultures were diluted three times per week (0.21 d^-1^). The P-sufficient cultures yielded algae with a C:P ratio that ranged between 150-230, and the P-limited cultures yielded algae with C:P ratios of 1000-1100.

### Statistical analyses

Because the infection develops with time, the statistical analyses were designed primarily to reveal interactions between time (day) and infection [11]. This was tested with population density, abundance of adults and juveniles, C biomass and individual sizes of adults and juveniles with GLM repeated measures ANOVA. Each food treatment was analysed separately. Homogeneity of variance and/or normality of residual requirements could not be met for some of the within-subject data even after transformation, but because ANOVA is robust against minor violations of these assumptions [57], and non-parametric tests (e.g., rank ANOVA) do not allow testing for interactions, we found that the statistics applied would be the most robust. In the analyses, day was included as a within-subject factor and infection status as a between-subject factor. After confirming a significant interaction between day and infection, we used the contrast of the within-subject factor to determine at what time point the changes in interaction occurred. The contrast used was the “difference”, which compared each level to the mean of all previous levels. The abundance of adults and juveniles was evaluated as a difference in total abundance at 8 d intervals, and further confirmation was provided by manual comparison to data obtained from ImageJ analysis. For analysis of population density and abundance of juveniles only values from day 12 onwards, when *Daphnia* started reproducing, were included, and for the abundance of adults, only values from day 20 onwards were used. Because the relative impact of infection on the population biomass between feeding treatments could not be compared statistically in a single test, we calculated the extent to which the infection changed the biomass in each feeding treatment in relation to the control populations. The relative biomasses for the infected populations for both feeding treatments were calculated by dividing the biomass of each infected population by the mean biomass of the control populations for each date (cf. [31]). The deviation of the mean relative biomasses for each day and feeding treatment from a reference value of one were tested with one-sample t-tests using Bonferroni corrections.

### Availability of supporting data

The data set supporting the results of this article is available in the Dryad Digital Repository http://dx.doi.org/10.5061/dryad.r50k9 [58].

## Additional file

Additional file 1:
**Schematic representation of the experimental setup for the population experiments.**
