# Supplementary material for: Phosphorus limitation enhances parasite impact: feedback effects at the population level
Source: BMC Ecol. 2014 Oct 31;14:29. doi: 10.1186/s12898-014-0029-1 (PMC4223164; doi:10.1186/s12898-014-0029-1)

Additional file 1. Schematic representation of the experimental setup for the population experiments.

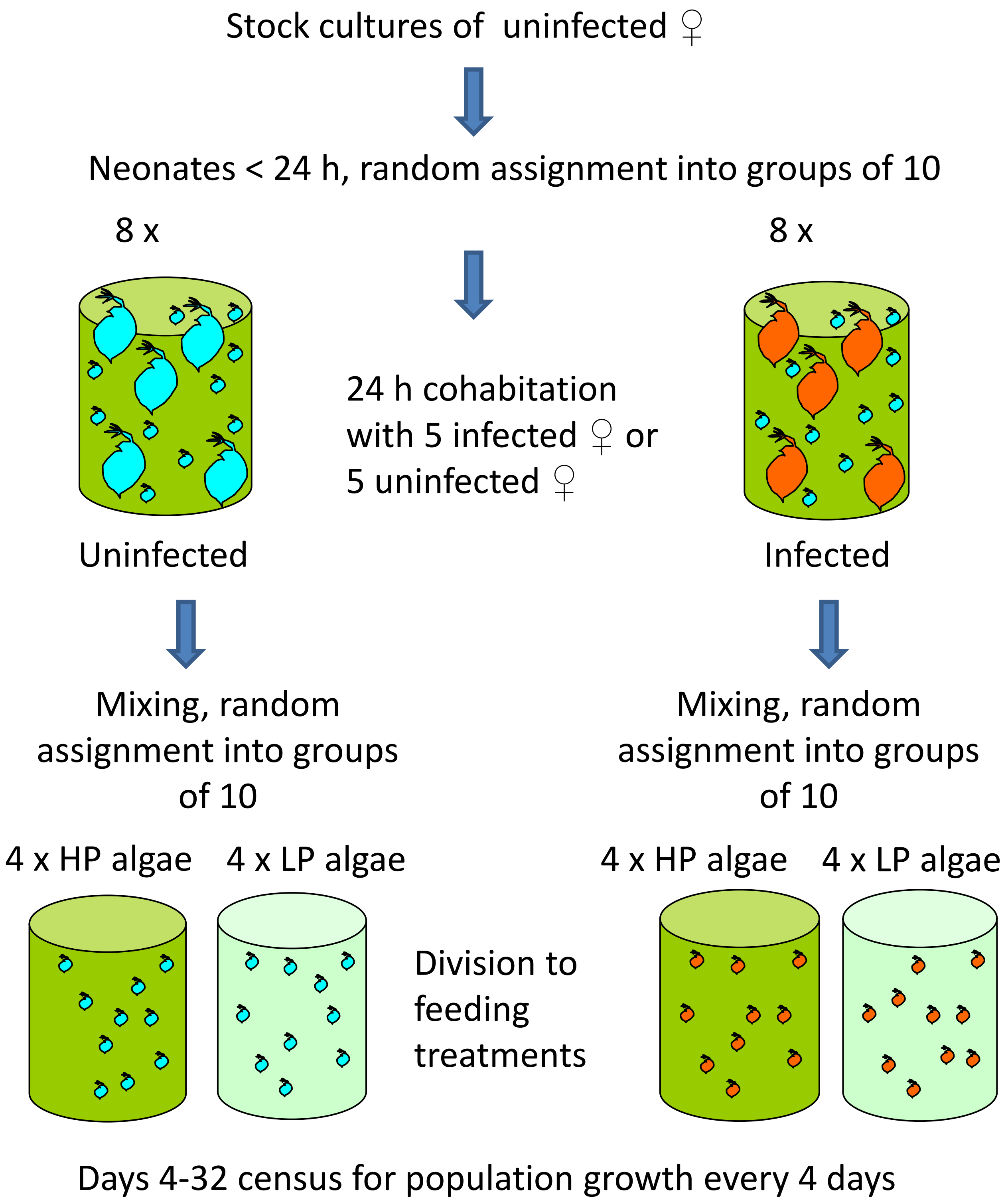

Supplement: Additional file 1: — Schematic representation of the experimental setup for the population experiments. [file 12898_2014_29_MOESM1_ESM.pdf]
